# Systematic review on raphide morphotype calcium oxalate crystals in angiosperms

**DOI:** 10.1093/aobpla/plad031

**Published:** 2023-06-06

**Authors:** Natasha S Lawrie, Nekane Medrano Cuetos, Francesca Sini, Ghifary A Salam, Hangyu Ding, Arthur Vancolen, Jessica M Nelson, Roy H J Erkens, Giuditta Perversi

**Affiliations:** Maastricht Science Programme, Faculty of Science and Engineering, Maastricht University, PO Box 616, 6200 MD Maastricht, The Netherlands; Maastricht Science Programme, Faculty of Science and Engineering, Maastricht University, PO Box 616, 6200 MD Maastricht, The Netherlands; Maastricht Science Programme, Faculty of Science and Engineering, Maastricht University, PO Box 616, 6200 MD Maastricht, The Netherlands; Maastricht Science Programme, Faculty of Science and Engineering, Maastricht University, PO Box 616, 6200 MD Maastricht, The Netherlands; Maastricht Science Programme, Faculty of Science and Engineering, Maastricht University, PO Box 616, 6200 MD Maastricht, The Netherlands; Maastricht Science Programme, Faculty of Science and Engineering, Maastricht University, PO Box 616, 6200 MD Maastricht, The Netherlands; Maastricht Science Programme, Faculty of Science and Engineering, Maastricht University, PO Box 616, 6200 MD Maastricht, The Netherlands; Maastricht Science Programme, Faculty of Science and Engineering, Maastricht University, PO Box 616, 6200 MD Maastricht, The Netherlands; Maastricht Science Programme, Faculty of Science and Engineering, Maastricht University, PO Box 616, 6200 MD Maastricht, The Netherlands

**Keywords:** Biomineralization, calcium oxalate, eudicotyledon, magnoliids, monocotyledon, phylogenetic distribution, raphides

## Abstract

Abstract. Calcium oxalate (CaOx) crystals are biominerals present in a wide variety of plants. Formation of these crystals is a biomineralization process occurring in vacuoles within specialized cells called crystal idioblasts. This process is dependent on two key components: deprotonated oxalic acid, and calcium ions (Ca^2+^), and can result in multiple crystal morphologies. Raphides are needle-like CaOx crystals found in various plant organs and tissues. Though their function is highly debated, they can potentially store calcium, sequester heavy metals, protect against herbivory and possibly programmed cell death. The last review of the taxonomic and anatomical distribution of raphides across the plant kingdom dates back to 1980, in a review by Franceschi and Horner, prompting an updated systematic review of raphides in plants. We conduct a broad literature search to record plant taxa and tissue locations containing raphides. We provide an overview of raphide-forming plant taxa, discussing phylogenetic distribution of raphides at the order level, and report on the specific locations of raphides within plants. Our review reveals raphide occurrence has been studied in 33 orders, 76 families and 1305 species, with raphides presence confirmed in 24 orders, 46 families and 797 species. These taxa represented less than 1 % of known species per family. Leaves are the most prominent raphide-containing primary location in all three major angiosperm clades investigated: Eudicots, Magnoliids, and Monocots. Roots are least reported to contain raphides. The collation of such information lays the groundwork to unveil the genetic origin and evolution of raphides in plants, and highlights targets for future studies of the presence and role of plant raphides.

## Teaser

Everyone knows crystals, for example, sugar crystals or ice crystals. Plants also contain crystals, made of a substance called calcium oxalate. These crystals can have different functions, such as protection against being eaten or redirection of light to help photosynthesis. One type of crystal is especially peculiar: the needle-shaped raphide. This type of crystal seemed to occur in a range of flowering plants groups. In this paper, we mapped the occurrence of raphides across plant groups onto an evolutionary tree and across the plant body (roots, leaves, stem and flowers). We found that the crystals indeed occur in many plant groups but are also strikingly absent in others. It is, however, unclear if they are really absent since these groups are often not investigated. Furthermore, how this particular crystal shape originates is unknown.

## Introduction

**Figure 1.**
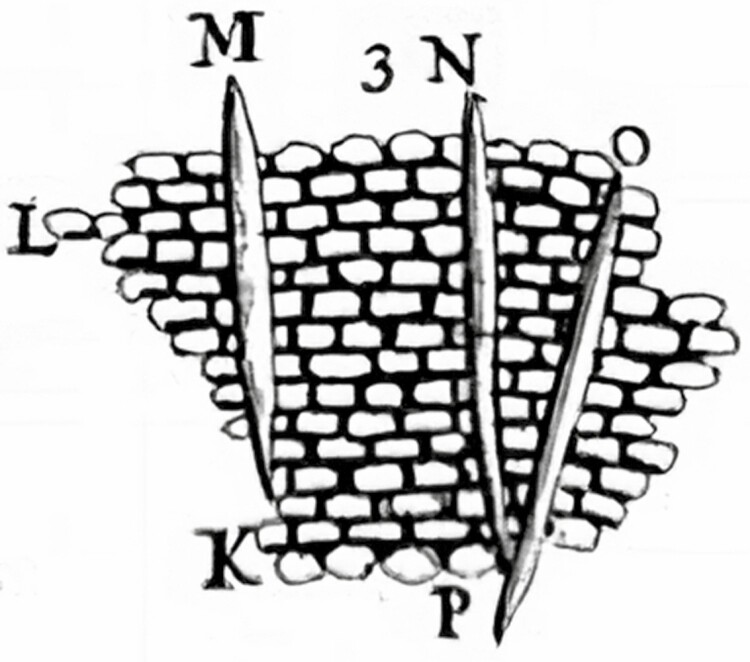
First depiction of calcium oxalate raphides by Antony van Leeuwenhoek (1705). At the state of the art, this drawing could also be depicting styloids crystals, as both morphologies are reported in *Cinchona officinalis*.

Reports of calcium oxalate (CaOx) crystals in plants date as far back as the 16th century, when light microscopy first allowed detailed observations of the anatomy of plant tissues. It is believed that the very first mention of these crystals is the description of ‘long particles’ whose ‘ends run into a point’ in a letter written by Antony van Leeuwenhoek in which he reported on the tissues of *china china*, or *Cinchona officinalis* as labelled by Linnaeus (Leeuwenhoek 1705; Lewis 1791). The needle-like crystals that van Leeuwenhoek reported (Fig. 1) were named ‘raphides’ by Dutrochet (1824) and De Candolle (1827). Both scientists, contemporary in their works, based the nomenclature on the Greek word for needle (Hess 1936). At present, CaOx crystals have been found in various plant tissues and organs of at least 215 families, and in approximately 74 % of angiosperm families (McNair 1932; Gallaher 1975; Zindler-Frank 1976; Franceschi and Horner 1980; Nakata 2012). It is now known that these crystals are formed by precipitation of deprotonated oxalic acid and calcium ions, and may occur in various hydration states and morphologies (Franceschi and Nakata 2005; Bauer *et al.* 2011).

Raphides are typically found in large numbers and can be all over the plant (Raman *et al.* 2014). The peculiar morphology of these crystals has led to many speculations about their specific function. However, there is no explanation yet for their unusual crystalline form and the biological pathways of their synthesis have yet to be elucidated. In this review, we investigate the available literature on plant-crystal biology and chemistry, and then subsequently focus on CaOx raphides. We document their occurrence in the plant body and their phylogenetic distribution. The results not only provide a state-of-the-art catalogue of raphide-forming plant taxa but also a first step towards understanding the origins and functions of raphide crystals. Our results provide a basis for predicting crystal presence in unstudied taxa by comparing them with closely related species. This will lay the foundation to ultimately unveil the evolution of raphides across the plant kingdom, from green algae to angiosperms. in order to understand whether raphide formation shows an evolutionary trend or is an ecological response to the environment.

## Review of Crystal Synthesis and Functions

### Biomineralization and biosynthesis

All plant crystals are the result of a biomineralization process. A biomineral is a hierarchically structured organic or inorganic material produced by a living organism, typically forming hard supporting structures, the most commonly known being bones, teeth and shells (Lowenstam and Weiner 1989; Crichton 2018). Vascular plants are capable of several biomineralization processes. The most common system is crystalline CaOx, with over 74 % of flowering plant families containing CaOx deposits (Nakata 2012). The second, more narrowly distributed biomineral type is calcium carbonate, in amorphous or crystalline form. Finally, calcium sulfate, calcium phosphate, magnesium oxalate, strontium oxalate, strontium and barium sulfate, as well as silicon dioxide in amorphous or crystalline form, have been observed in some plant species (Arnott 1982; He *et al.* 2014).

Formation of biominerals may be biologically induced or biologically controlled. Biologically *induced* mineralization occurs when an organism’s activities, such as metabolism, diffusion or building cell walls, incidentally create conditions that favour mineral precipitation (He *et al.* 2014; Chen *et al*. 2019). In this case, the organism has little to no control over the localization, orientation, morphology or composition of the crystal formed (He *et al.* 2014). Biologically *controlled* mineralization, on the other hand, is caused by purposeful biological activities of the organism itself, and occurs in delineated and confined spaces (Chen *et al.* 2019), thus controlling the localization, shape and size of the crystals (He *et al.* 2014). The formation of CaOx raphides is a case of biologically controlled biomineralization.

The two key chemicals involved in CaOx biosynthesis are calcium and oxalic acid. Vascular plants acquire calcium by osmosis from the soil through their roots, and distribute it throughout their tissues (White 2001). It is presumed that Ca²^+^ circulates mainly via the apoplastic pathway in the root cortex, although the relative contributions of the apoplastic and symplastic pathways to the xylem are unknown (White 2001). Once in the endodermis, Ca²^+^ is transported through the xylem and deposited locally. Beyond general metabolism, calcium is also used by plants for synthesis of organelles and as a key element for various signalling pathways (Marinos 1962). Since excessive calcium concentrations are cytotoxic, its transport in the phloem is strictly regulated. Excessive Ca²^+^ ions in cells are sequestered and stored as insoluble salts of oxalic, phosphoric and phytic acids (Franceschi and Horner 1980).

**Figure 2.**
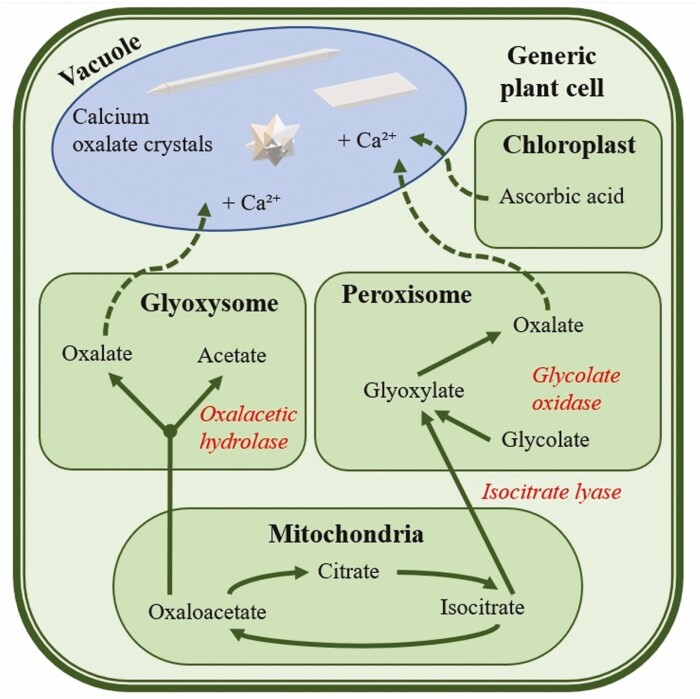
Simplified metabolic pathways of oxalic acid production in plants. Adapted from Franceschi and Horner (1980).

On the other hand, oxalic acid is synthesized by the plant itself (Franceschi and Loewus 1995) through multiple processes that are summarized in Fig. 2. Even at the neutral pH of the cellular environment, its high acidity all but ensures its deprotonation. We can therefore always consider that after production it will be present as oxalate anions in the plant cell (Li *et al.* 2022). Oxalic acid can be formed in the peroxisome through the oxidation of glyoxylate and glycolate (Seal and Sen 1970), or via the hydrolysis of oxaloacetate in mitochondria (Franceschi and Horner 1980). However, multiple studies demonstrate that those methods of production of oxalic acid may not be the main source responsible for the crystal formation (Zindler-Frank 1976; Kausch and Horner 1985; Zindler-Frank and Horner 1985). A consensus has not yet been reached, but Franceschi and Horner (1979) demonstrated that the major source of oxalic acid responsible for crystal growth was ascorbic acid, which is found in all plant tissue at a relatively high level. This approach has the support of subsequent studies based on radiolabelling and microautoradiography techniques (Horner *et al.* 2000; Kostman *et al.* 2001; Debolt *et al.* 2007).

CaOx formation most often occurs inside the vacuoles of specialized cells called crystal idioblasts (Foster 1956). The distinctive characteristics of crystal idioblasts include an enlarged nucleus; specialized plastids known as crystalloplastids that do not contain thylakoids, grana or RuBisCO and lack photosynthetic activity (Li and Franceschi 1990); high ER and rRNA content and unique vacuolar components and membrane structure (Arnott and Pautard 1970; Horner and Whitmoyer 1972; Horner and Wagner 1995).

**Figure 3.**
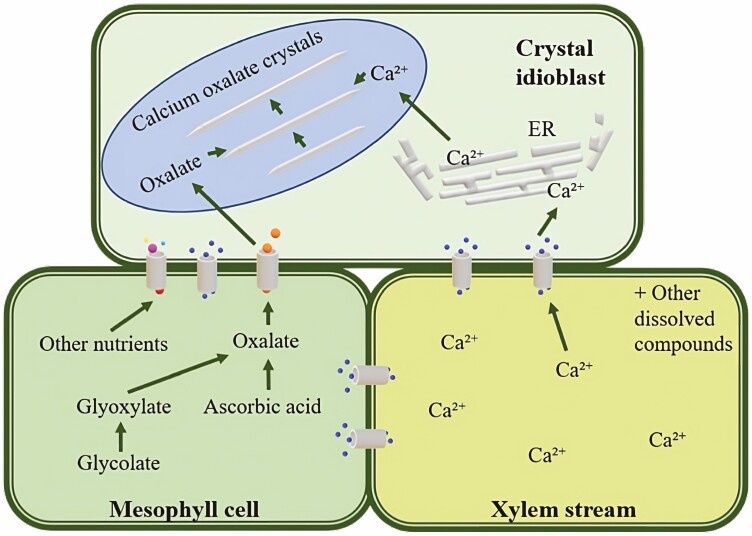
Model of CaOx formation in idioblasts. Adapted from Franceschi and Nakata (2005).

The initial steps of crystal formation have been found to involve non-crystalline elements acting as a matrix to aid in the further crystal formation (Horner and Whitmoyer 1972; Tilton and Horner 1980; Arnott 1982; Li *et al.* 2003). CaOx formation can be summarized in a few steps as shown in Fig. 3. Firstly, calcium enters the cell via the apoplast using the xylem stream, and the ions in excess gradually accumulate in the idioblasts. The free calcium ions are then taken up by the ER, and at the same time, oxalic acid is produced by nearby cells. Both calcium and oxalic acid are subsequently transferred to the vacuole of the idioblast, where they move through the membrane and are added along the newly formed crystal. While the exact mechanism for the calcium and oxalate transfer is not clear, the vacuoles of crystal cells are reported to accrue several substances with strong Ca-binding (Karabourniotis *et al.* 2020). In particular, raphides are surrounded by a complex polysaccharide-containing mucilage (Kausch and Horner 1984; Wang *et al.* 1994; Webb *et al.* 1995). These substances might have a role in facilitating exchange in the crystal cells between the cytoplasm and the vacuole, thus directing the passage of calcium (Webb 1999).

In the vacuole, organic paracrystalline bodies form the membrane crystal-chambers in which the CaOx crystals develop (Frank and Jensen 1970; Franceschi 1984). The composition of these membranes is largely unclear, but they are suggested to play a role in the control of crystal formation (Arnott and Pautard 1970; Franceschi 1984; Webb 1999). The functionality of the antigenic proteins on the crystal-chamber membranes is also not fully elucidated. In *Yucca* roots, the double-pointed raphide deposition and growth occurs at both ends (Horner *et al.* 2000). Notably, the presence of paracrystalline bodies associated with membranes and crystal chambers has been shown for not only for raphides but also for druses and crystal sand (other studies not included; see Horner and Wagner 1995).

The mechanism beyond crystal formation is not only a question of transportation of calcium and oxalic acid into the vacuole but also of the tight coordination between crystal growth and cell growth. Kostman *et al.* (2003) showed that when the growth of the cell is inhibited, the crystal growth is also affected and stops to avoid piercing the vacuole. Once the crystals are fully grown, the addition of calcium and oxalic acid is stopped. At this point, the cell is fully mature and stays in this configuration until cell death.

### CaOx crystal morphology

**Figure 4.**
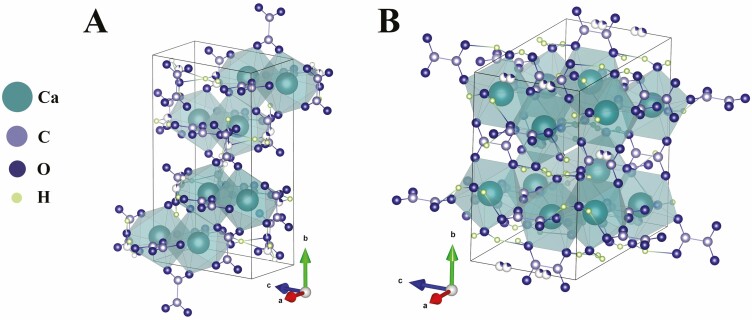
(A) Crystalline structure of monohydrate whewellite. (B) Crystalline structure of dihydrate weddellite. Arrows indicate the orientation of the lattices. Atoms are colour-coded according to the legend, note that for some oxygen atoms partial colouring correlates to a partial occupancy of the crystallographic site. Structure adapted from Izatulina *et al.* (2018). Created with Vesta plotting program (Momma and Izumi 2011).

As a mineral, CaOx can form with different amounts of water molecules trapped into the structure, leading to different crystals depending on the hydration state. The most commonly found in plants are whewellite (CaC_2_O_4_·H_2_O, monohydrate) and weddellite (CaC_2_O_4_·2H_2_O, dihydrate), based on their thermodynamic stability. Whewellite crystallizes in a monoclinic space group (Fig. 4A), whereas Weddellite adopts a more highly symmetrical tetragonal configuration (Fig. 4B; Frey-Wyssling 1981). In mineral form, crystals of CaOx can naturally be found at varying levels of hydration between 1 and 2 without affecting the crystal structure (Sterling 1965). While the monohydrate form is the most thermodynamically stable, and the most commonly found in plant biomineralization, higher hydration states are also reported (Arnott *et al.* 1965). The hydration state relates to the condition of biosynthesis and it can be a unique key, not only to interpret the morphological features of these crystals but especially to address the metabolic and transport pathways that lead to their formation in plants (Webb 1999).

Calcium oxalate crystals have been reported to appear in plants in five distinct morphologies: raphides, druses, prisms, styloids and crystal sands (Raman *et al.* 2014). In some instances, crystals have been classified as whewellite or weddellite solely on the basis of their shape. However, recent evidence indicates that crystal shape might be independent of the hydration state of CaOx (ICPT 2019). It could therefore be possible for the biomineralization pathways to be the distinguishing feature between members of different families: an underlying genetic control might affect the capability to synthesize CaOx in varying hydration states, therefore resulting in one or more morphotypes taking prevalence.

**Figure 5.**
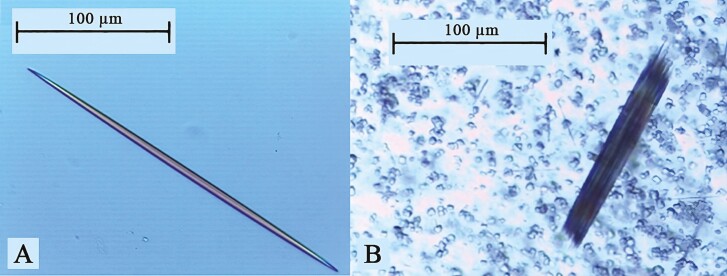
Optical microscopy image of (A) a thick raphide and (B) thick raphides organized in a bundle. Both samples have been extracted and isolated from Aloe vera mesophyll cells (photographs by A. Vancolen).

Raphides are distinguished by their pointed needle-like shape and range in length from around 16 up to 300 μm in some plants (Cote 2009; Chu *et al.* 2014). Raphides are typically observed in large numbers and closely packed and arranged in parallel in regular cylindrical bundles (Cote 2009; Raman *et al.* 2014), as shown in Fig. 5. This arrangement characterizes both thicker raphides, which are commonly found in leaves and are arranged in bundles approximately 23 μm long, as well as thinner raphides, which are typically seen in reproductive organs (Cote 2009).

**Figure 6.**
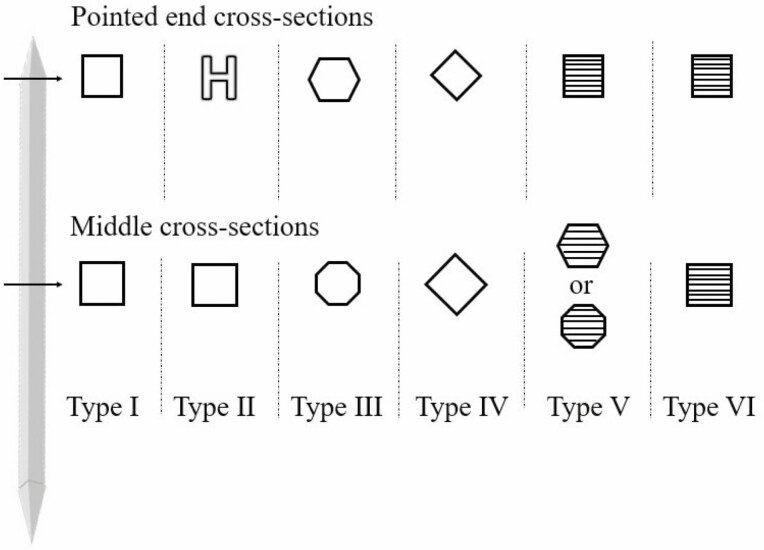
Schematic representation of the six types of raphide crystals, showing cross-sectional views. Note the crystalline sheets in the cross-sections of Type V and VI crystals. Adapted from Raman *et al.* (2014).

Six different types of raphide crystal morphologies have been reported by Raman *et al.* (2014), expanding on the four previously reported (Horner and Wagner 1995), and are ­represented in Fig. 6. Type I crystals are characterized by a four-sided cross-section (identified in *Psychotria*). Type II crystals appear, similar to type I, with a four-sided square in the middle cross-section, yet towards each end two opposing grooves giving an ‘H’ shape are present (identified in *Lemna*). Type III needles are pointed at both ends and possess six-to-eight sides, yielding a hexagonal or octagonal cross-section (identified in *Agave*). Type IV raphide crystals have four sides with a median division and are pointed at one end and notched at the other end (identified in *Vitis*). Type V crystals are observed to be six-sided, rarely eight-sided, around 125 to 160 μm long, and pointed at both ends whose cross-section is four-sided. Finally, type VI crystals are distinguished by a four-sided cross-section with bevelled ends, measuring 130 to 165 μm long (Raman *et al.* 2014).

This level of variability is a remarkably unique feature compared to other biocrystals, but this in-depth morphological analysis is not found in the available literature beyond the work of Raman *et al.* While it is suggested that some of these types could be variants of existing types of crystals (Type V related to Type III, Type VI related to styloids) and others could be interchanged with the level of oxalate hydration (Type V and Type VI, with their peculiar crystalline sheets), none of these hypotheses have been biologically and crystallographically systematized at the moment.

### CaOx crystal functions

It is believed that calcium regulation is one of the main functions of CaOx biocrystals. The presence of calcium is integral to the growth and health of plants, but can be toxic when accumulated in excess (White and Broadley 2003). Numerous studies have discovered variations in the number and size of CaOx crystals in response to changing calcium levels (Franceschi and Horner 1979, 1980; Franceschi 1989). However, not all morphologies are equally susceptible to reversible calcium sequestration. For instance, raphides, given their large size and stability, are less likely to act as regulatory elements (Volk *et al.* 2002).

Secondly, it has been speculated that druses and prisms may aid in photosynthesis optimization. Druse crystals are generally found in the palisade cells located in the upper regions of leaves, suggesting a common function across species (Gal *et al.* 2012). A series of experiments by Pierantoni *et al.* (2017) concluded that when light is directed onto the area high in druse content, reemission occurs with a much lower intensity and larger scattering as compared to the areas with a lower amount of druses. These results convey that druses play a role in photosynthesis by scattering light so that it can easily be received by nearby photosynthetic cells. It is possible that the common placement in the upper palisade may simply indicate a dependence of their synthesis based on features of the environment (i.e. high light availability), although it has also been observed that in a low-light-adapted plant, *Peperomia glabella*, druse crystals exhibit an adaptive response to differing light conditions. Namely, the position of druse crystal changes within the palisade cell and druse crystal diameter decreases, with more intense light (Kuo-Huang *et al.* 2007). Another photosynthesis-related role in leaves might be ‘alarm photosynthesis’, where large CaOx crystals act as biochemical reservoirs of non-atmospheric carbon. In *Amaranthus hybridus*, CaOx crystal abundance showed a gradual decrease during daytime and total recovery during the night. Under drought conditions, where stomatal closure is preferred to prevent excessive evaporation, the crystals in the mesophyll were decomposed by oxalate oxidase and converted into CO_2_. Under the same conditions, similar results were obtained with *Dianthus chinensis*, *Pelargonium peltatum* and *Portulacaria afra*. The crystal degradation observed during the day provides supplemental carbon for photosynthetic assimilation, particularly under drought conditions (Tooulakou *et al.* 2016). However, not much is known about this relatively new photosynthetic pathway.

Thirdly, CaOx crystals are hypothesized to be associated with plant tolerance mechanisms against toxic heavy metals in a similar fashion as with excess calcium ions (Nakata 2003; Franceschi and Nakata 2005). For instance, aluminium ions and oxalic acid have been found to be compounded by plants to produce non-toxic crystals. Heavy metals, such as lead and aluminium, are found in the majority of agricultural soils but have detrimental effects on plants, including reduced growth, altered metabolism and reduced crop yield (Goyal *et al.* 2020). Cellular damage can occur due to uncontrolled induced oxidative stress, along with disruption of cellular ionic homeostasis (Yadav 2010). Moreover, plants use programmed cell death (PCD) as a normal part of cell turnover. The decomposition of CaOx produces one molecule of hydrogen peroxide (H_2_O_2_) per oxalate degraded (Karabournioutis *et al*. 2020). As this molecule can be a trigger of cell death, CaOx might represent an oxalate pool that can provide H_2_O_2_ to be used in PCD (Caliskan and Cuming 1998). Raphides and other crystalline structures are implicated in PCD (e.g. in Typha, Capsicum anthers, etc.). Some aquatic plants such as *Nymphea* and *Myriophyllum* utilize special crystal cells in young tissues that hold druses and prisms for PCD (Ni *et al.* 2014; Du *et al.* 2018).

There are also indications that CaOx can be involved in cell wall strengthening with lignin (Caliskan and Cuming 1998), a process that ultimately also leads to the death of cells.

Finally, CaOx crystals, primarily raphides, have been proposed to be a defence against herbivores, both in active and passive forms. Active defences refer to the harm inflicted on the herbivore upon the mechanical penetration of crystals into tissues. Thurston (1976) described grooved raphide crystals found in the stinging cells of *Tragia ramosa* that would be forcefully expelled into tissues during ingestion or attack. It was further suggested that the grooves contain alkaloid toxins that can reach the tissues and cause further irritation (Sakai and Hanson 1974; Thurston 1976). The combined effect between groove morphology and production of biological toxins elucidates a ‘needle-effect’ of raphides, strengthening the action of the toxin by penetrating through tissue (Konno *et al.* 2014). Although the toxins that act synergistically with raphides are largely unidentified, the authors proposed that they could include proteinaceous molecules including cysteine proteases. Conversely, passive defences, the ability of the plant to minimize the effect of an attack due to physical and chemical factors (Van der Westhuizen and Bornman 2004), may also deal with the structure of the crystals. Korth *et al.* (2006) found that caterpillar larvae of *Spodoptera exigua* would prefer to feed on mutant lines of *Medicago truncatula* leaves that had reduced amounts of prismatic CaOx crystals rather than on the wild-type leaves. The researchers proposed that this is due to the abrasive effects caused by the prismatic crystals on mandibles of the larvae. Furthermore, it was shown that in different stages of the banana and plantain fruit (Musa L. cultivars), the presence, size, and abundance of raphide CaOx crystal would change, indicating a functional predisposition to protect unripe and young fruit against herbivory (Osuji and Ndukwu, 2005). However, a recent commentary opposes the notion that raphides act as an effective herbivory defence mechanism (Paiva 2021). Paiva (2021) acknowledges that CaOx crystals cause distinguishable mechanical damage to some herbivores, influencing deterrence and avoidance patterns, but the defence provided by CaOx crystals ‘may be overstated’, given that assertions have mostly been made based on their morphologies and on their hard and indigestible nature, rather than experimental evidence. Despite all of the aforementioned functions, in the *M. truncatula* species CaOx crystal formation has found to be non-essential, in itself, for plant growth or development (Nakata and McConn 2003).

## Methods

A systematic literature review was conducted in order to build a comprehensive resource on raphide-containing plant taxa. Articles were collected through the search function in Web of Science (Clarivate Analytics) across all databases, using the keyword raphide*. The indexing service was used as it offers an extensive list of relevant articles, while the keyword *raphide* was sufficient to isolate the relevant literature, since this crystal morphology is exclusive to CaOx.

The following selection criteria were enforced on the literature results:

(a) The paper must have studied or reviewed studies on *plants* of any family and must have reported *species* names.
(b) The paper must have reported the presence of *calcium oxalate raphides* within the plant.
(c) The paper either (i) reported the *location* of the plant organ where the raphides were found (i.e. leaf, reproductive organ, root and stem,) or (ii) did not report the location, to which the species was counted as a raphide-forming species in preliminary analysis, but excluded from the later locations analysis.
(d) Only primary resources for raphide reports were used to avoid double counting of reports and citations.

**Figure 7.**
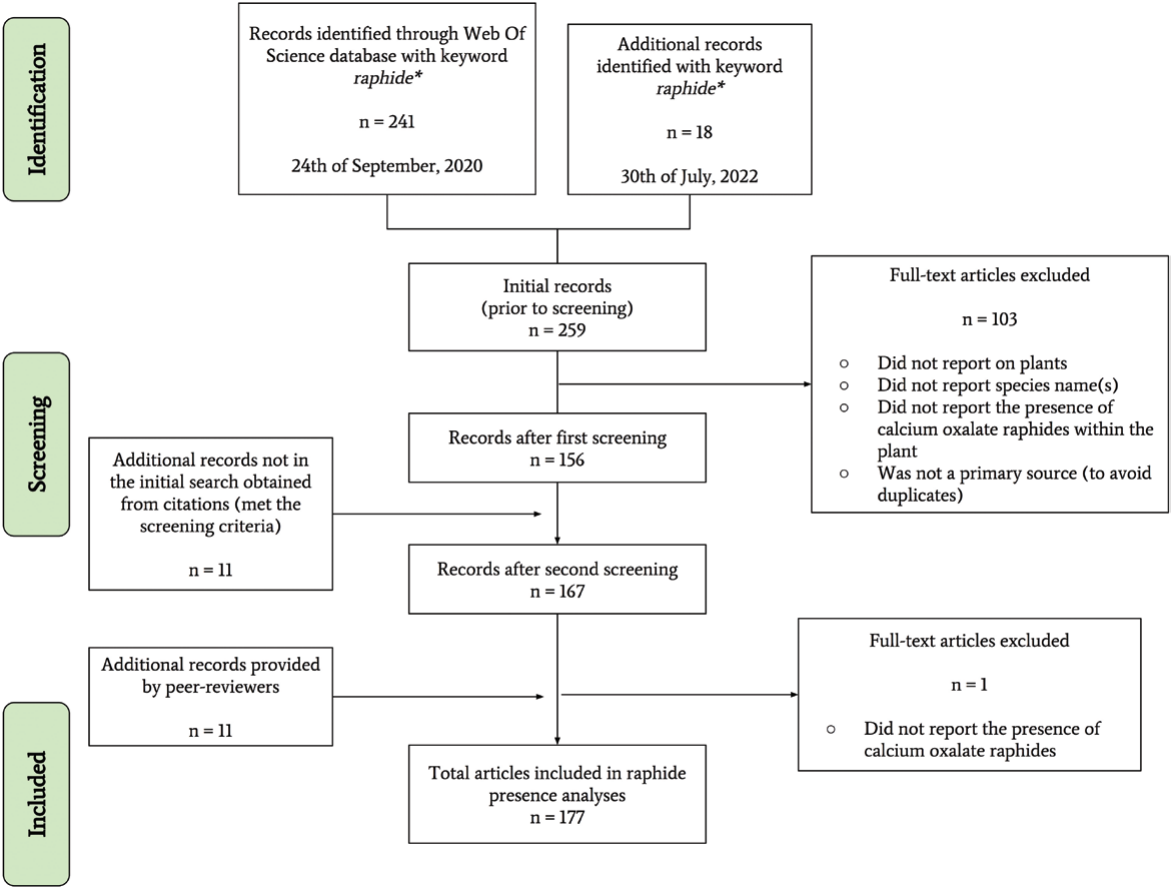
Preferred Reporting Items for Systematic reviews and Meta-Analyses (PRISMA) flowchart for this review paper, including revisions and peer-review amendments and integrations.

The flow of identification, screening and inclusion is summarized in Fig. 7, and resulted into a collection of 177 papers (repository available at the provided link **Supporting Information—Link S1)**.

Information on the location of raphides in the plants was extracted **[**see **Supporting Information—Table S1****]**. Notably, different papers utilized different terminology, and thus, there were disparities between the named plant structures, tissues or cell types discussed. Here, the locations were divided into the four generally accepted parts of a plant (primary locations): leaf, reproductive organ, stem and root [see **Supporting Information—Table S2**]. Some species showed raphide presence in more than one primary location (e.g. leaf and stem). For analysis of raphide distribution in plant organs at a species rank, species were counted more than once if raphides were present in more than one primary location, i.e. if raphides were recorded in leaves and stems of a species, it was counted in both categories. A further subdivision was made based on the specified locations obtained directly from the literature (Table 1). Such secondary locations may belong to more than one primary location, such as parenchyma (both leaf and root) or aerenchyma (both stem and root) **[**see **Supporting Information—Table S2****]**.

**Table 1.** Summary of primary and secondary location categories. The difference in terminology and specificity is dependent on the source (see main text). If secondary locations were not specified in the paper, they were noted as ‘unspecified’.

Leaf	Reproductive organ	
Aerenchyma, air canals, chlorenchyma, colleter, cortex, epidermis, lamina, mesophyll, parenchyma, petiole, pinnae, trichomes, unspecified, vascular	Androecium	Anther, pollen, stamen
	Gynoecium	Ovary, stigma, style
	Embryo, flower, flower bud, fruit, meristem, pseudopedicel, seed, petal, sepal, unspecified	

Root	Stem	
Aerenchyma, central cylinder, cortex, exodermis, meristem, parenchyma, primary roots, root, apex, root tuber, unspecified	Bark, callus, collenchyma, corm, cortex, epidermis, ground tissue, meristem, parenchyma, pseudostem, rachis, sclerenchyma, tuber, tylose, unspecified, vascular, wood	

Species for which no raphides were found, or for which no anatomical location was presented, were excluded from the analysis of location occurrence. These species (i.e. non-presence reports) were nonetheless included in the analyses of phylogenetic distribution.

All taxon names were verified according to Erkens (2011) using the Plants of the World Online website (POWO 2019) and the International Plant Names Index (IPNI 2020). Synonyms were updated to their accepted name **[**see **Supporting Information—Table S1****]**.

Progress of raphide research in plant taxa was summarized as a percentage of species in a taxon studied for raphide presence (number of species investigated for raphides/total number of species in taxon). Coverage of taxonomic groups was summarized by calculating the percentage of studied species in the taxon in which raphides have been detected (number of species in taxon with raphides detected/number of species investigated for raphides).

Results at the order level were plotted using the Interactive Tree of Life web tool (Letunic and Bork 2021), based on the most recent phylogenetic tree, as found on the Angiosperm Phylogeny Group Website (Stevens 2001 and onward) with ferns and gymnosperms included as sister groups.

## Results

### Phylogenetic distribution of raphides

**Figure 8.**
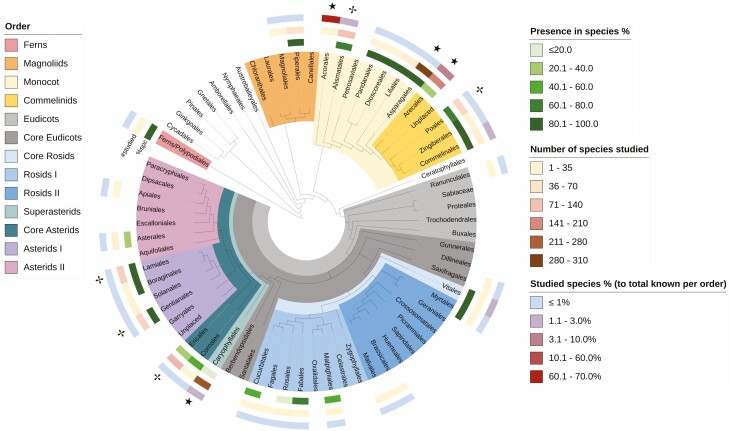
Phylogenetic tree showing the distribution of raphides across vascular plants. Number of species studied within an order and the percent ratio of the number of studied species to the total known in an order are marked by coral and blue–red coloured markers respectively, with colour saturation denoting the count and percentages. Orders for which the sampling numbers are relatively high (>150 species) or highly representative of the order members are additionally marked with full stars (⋆), while those which have been considerably represented or sampled (50–150 species) are marked with crosses (✢). Raphide occurrence and percentage of raphide presence in studied species within each order are shown by green markers, with colour saturation denoting the percentage. Any unmarked bar associates to lower sampling. The tree was generated using the Interactive Tree of Life (IToL) web tool (Letunic and Bork 2021).

We found 33 orders, 76 families and 1305 species that have been investigated for raphide presence. Among these, 24 orders, 46 families and 797 species have evidence of raphides present (Fig. 8). All of these are vascular plants and the vast majority are angiosperms. Of the 33 orders investigated, 29 have data for 50 % or less of their known families. The ­majority of families are severely undersampled for raphide presence (i.e. represented by under 1 % of known species). A complete overview can be found in the spreadsheet in **Supporting Information—Table S3**.

Out of the 33 orders studied, only Acorales, Alismatales, Arecales and Caryophyllales are represented by more than 2 % of their known species (66.7, 2.1, 7.9 and 2.3 % respectively). These orders have low percentages of crystal-forming species, ranging from 0 to 34.2 %, except for Alismatales with 76.6 % of investigated species forming raphides. Aside from Acorales with only 2 samples, these orders also have high species coverage numbers, with counts for Alismatales, Arecales and Caryophyllales being 94, 190 and 264 samples respectively. All remaining orders are represented by <2 % of known species. Despite the low percentage, the orders Asparagales, Poales, Ericales, Gentianales and Lamiales have been covered considerably (with 305, 57, 114, 61 and 73 individual samples respectively) compared to the remaining orders with less than 35 species. To highlight research trends and bias, the orders were marked into two groups based on their representation percentage and species coverage, as seen in Fig. 8. Acorales and Asparagales are marked as highly studied (denoted by the star ⋆ mark) as they have drastically high representation (Acorales) and sampling coverage (Asparagales). Arecales and Caryophyllales are also considered highly studied, having both >2 % representation and >150 species coverage. Alismatales, Poales, Ericales, Gentianales and Lamiales are grouped as moderately studied (denoted by the cross ✢ mark) because of their considerable representation or coverage, although not as high as the previously noted orders.

The highest percentage of raphide-forming species were found in these orders; Boraginales, Commelinales, Dioscoreales, Lamiales, Liliales, Myrtales, Pandanales, Polypodiales, Piperales, Vitales and Zingiberales, with 100 % of investigated species showing raphide presence. Though it is important to take into account that these orders have a low percentage of representative species, i.e. only 15 out of 808 species have been investigated for raphides in Commelinales (1.7 %), but all 15 species show raphide presence (100 %). However, not all orders with low representative species have high raphide presence in investigated species, i.e. 114 species out of 10 364 known Ericales species (1.0 %) were investigated for raphides, with only 37.7 % of species investigated forming raphides.

Notably, 30 families across 15 orders present no evidence for raphide formation. However, 14 orders, including Apiales and Brassicales, are represented by <0.1 % of known species, except for Acorales (66.7 %).

Eight families are represented by more than 10 % of known species: Acoraceae (66.7 %), Cactaceae (13.2 %), Philydraceae (20.0 %), Cyrillaceae (50.0 %), Marcgraviaceae (31.5 %), Roridulaceae (50.0 %), Sarraceniaceae (12.5 %) and Tetrameristaceae (60.0 %). Out of these, Acoraceae, Cyrillaceae and Sarraceniaceae contain no crystal-forming species. Only 13.9 % of Cactaceae species showed raphides, as opposed to 39.0 % of Marcgraviaceae, or 100 % of Philydraceae, Roridulaceae and Tetrameristaceae species. Interestingly, despite the representative percentage of species per family being 1 %, 255 out of 257 Orchidaceae species have been shown to contain CaOx raphides (99.2 %). It is thus probable that this is a characteristic trait of the family. Similarly, 96.6 % and 100 % of sampled Rubiaceae and Oleaceae species, respectively, were reported to have raphides (though Rubiaceae and Oleaceae only have 0.4 and 8.9 % species representation respectively).

### Raphide distribution in plants organs

**Figure 9.**
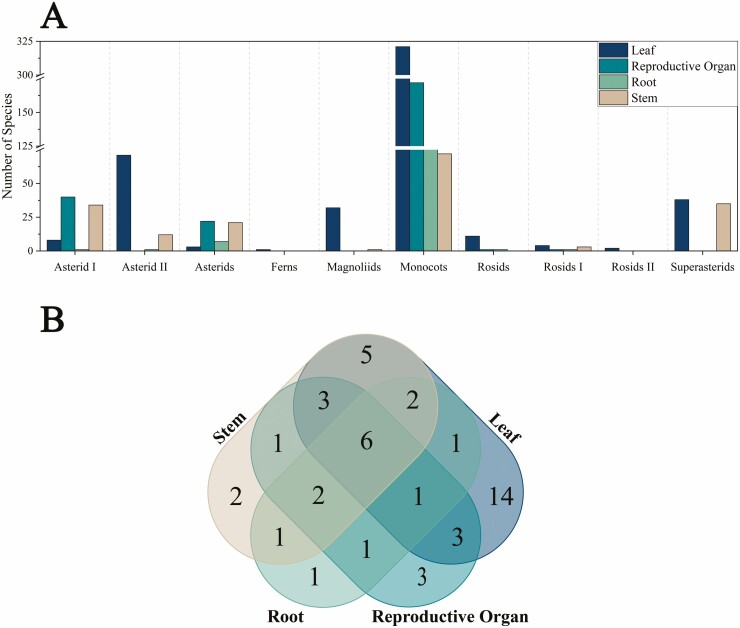
(A) The presence of raphides in each primary location, clustered by clade, for a total of 786 unique species. For Asterids, Superasterids and Rosids, nested clades are highlighted with their unique names; orders in these larger clades outside of subclades are listed under the main name. (B) Venn diagram showing the overlap of families containing raphides in each of the four primary locations: leaf, stem, root and reproductive organ.

The location of raphides was reported in 167 articles, representing 797 unique species across 46 families and 24 orders (Fig. 9A; **Supporting Information—Table S4**). Out of the four primary locations, leaf, reproductive organs, root and stem, the most common location in which raphides are present is the leaf (491 species), with all clades displaying at least 50 % raphide presence in leaves except for Rosids (33.3 %), Asterids (7.1 %), and Asterids I (13.8 %). These percentages are based on the number of species with raphides present in a certain primary location (in this case, the leaf) over the total number of species studied in literature, including those that gave a negative outcome for raphide crystals. Therefore, percentages are skewed by the varying coverage of studied species. For example, 100 % of species within Rosids II are positive for raphides in the leaves, but this percentage is based on two species out of the total five studied in various sources. Comparatively, Asterids I are reported with 8 species with raphides present in the leaves out of 58 species sampled which produces a percentage of 13.8 % species with raphide present in the leaves. This means that although Rosids II have a higher percentage of raphides in leaves, it does not necessarily mean it is a clade-wide pattern, as it has a low-species representation. Beyond the leaves, CaOx crystals are then most commonly found in the reproductive organ, with 236 species reported to have crystals within that primary location. Whereas Asterids and Asterid I had a low proportion of species with CaOx crystals in their leaves, these clades actually show the highest proportion of raphides in the reproductive organ out of all other clades, with 52.4 and 69.0 % respectively. This means that 22 out of 42 Asterids and 40 out of 58 Asterid I species have raphides in their reproductive organs. Contrastingly, whereas all clades had raphide presence in leaves, only five clades show a raphide presence in reproductive organs; Monocots (36.6 %), Rosids (7.7 %), Rosids I (12.5 %), Asterids (52.4 %) and Asterid I (69.0 %). The rest of the clades, Ferns, Magnoliids, Rosids II, Superasterids and Asterids II, do not show any presence of CaOx crystals in the reproductive organ. The stem shows a total of 178 species, of which three clades do not have any raphides present, namely, Ferns, Rosids and Rosids. Two clades have a raphide presence in stems greater than 50 %; 92.1 % for Superasterids and 58.6 % for Asterid I, where else all other families show raphide presence in stems as less or equal to 50 %; Magnoliids (3.1 %), Monocots (13.6 %), Rosids I (37.5 %), Asterids (50 %) and Asterid II (16 %). One hundred and thirteen species were found to have raphides in the root, with only six representative clades, Monocots (19.3 %), Rosids (7.7 %), Rosids I (12.5 %), Asterids (16.7 %), Asterid I (1.7 %) and Asterid II (1.3 %), as the other clades had no evidence of raphide presence. The Monocots clade is consistently the most represented for each primary location by count as it is the most studied, and also contains one species in which raphides were found in all primary locations examined, namely *Agave tequilana*.

Several species have raphides in more than one primary location, e.g. *Selenicereus hamatus* (Cactaceae) has raphides in the stem and leaf. A further breakdown of the raphide presence in primary location on a family basis allows for the identification of some more specific trends of occurrence (Fig. 9B; Table 2). The most common locations are the leaves, followed by the reproductive organs, stems, and finally roots. In 13 families, raphides are reported in leaves only. Similarly, three families only have raphides in the reproductive organs, namely, Passifloraceae (Rosid I), Philydraceae (Monocots) and Roridulaceae (Core Asterids). Conversely and interestingly, six families contain raphides in all four primary locations: Araceae, Arecaceae, Asparagaceae, Orchidaceae and Commelinaceae, which are all Monocots, and Rubiaceae, which belongs to Asterids I. The next overlap is between leaves and stems, as observed in Piperaceae (Magnoliids), Lowiaceae (Monocots), Fabaceae (Rosids I), Cactaceae (Superasterids), and Acanthaceae (Asterids II). A double overlap of leaves and reproductive organ categories is found in three Monocots families (Haemodoraceae, Liliaceae and Bromeliaceae), and a triple overlap of leaves, stems, and reproductive organs can be identified with three Monocots (Dioscoreaceae, Heliconiaceae and Musaceae). As can be seen, Monocots contain raphides in a wider array of locations when compared to the other clades.

**Table 2.** Occurrence of raphides in different primary locations, grouped by family. The table complements the Venn diagram in Figure 9B and shows occurrence of increasing overlap from left to right (i.e. raphides reported in one location, two locations, three locations or four locations simultaneously).

**Leaf**	**Leaf + reproductive organ**	**Reproductive organ + root + stem**	**Leaf + reproductive organ + root + stem**
• Lamiaceae • Boraginaceae • Amaryllidaceae • Aspleniaceae • Aizoaceae • Hydrangeaceae • Onagraceae • Rosaceae • Alstroemeriaceae • Nyctaginaceae • Cyclanthaceae • Typhaceae • Oleaceae • Euphorbiaceae **Reproductive organ** • Roridulaceae • Passifloraceae **• **Philydraceae **Root** **• **Colchicaceae **Stem** • Cucurbitaceae **• **Tetrameristaceae	• Liliaceae • Haemodoraceae • Bromeliaceae **Leaf + root** • Asphodelaceae **Leaf + stem** • Lowiaceae • Acanthaceae • Cactaceae • Fabaceae **• **Piperaceae **Reproductive organ + stem** • Marcgraviaceae **Reproductive organ + root** **• **Actinidiaceae	• Pontederiaceae • Ebenaceae **Leaf + root + stem** • Pandanaceae **• **Dioscoreaceae **Leaf + reproductive organ + stem** • Musaceae • Balsaminaceae • Heliconiaceae **Leaf + reproductive organ + root** • Vitaceae	• Commelinaceae • Orchidaceae • Araceae • Rubiaceae • Arecaceae • Asparagaceae

A more detailed breakdown was based on secondary categories was created (Table 1; **Supporting Information—Table S5**). The predominant secondary location for leaf is the parenchyma, whereby the proportion of species to report parenchyma in comparison to another secondary location is calculated as 41.4 %. This is followed by unknown (9.9 %), and the mesophyll (9.3 %). For the reproductive organ it is the gynoecium (33.3 %) followed closely by androecium (25.1 %). The predominant secondary location for the root is the cortex (32.6 %), followed by unknown (25.4 %) and root tuber (18.1 %). Secondary locations are mostly not reported for the stem (i.e. the most prominent category is ‘unspecified’), but the most abundant location after unknown is the stem cortex (19.9 %).

**Figure 10.**
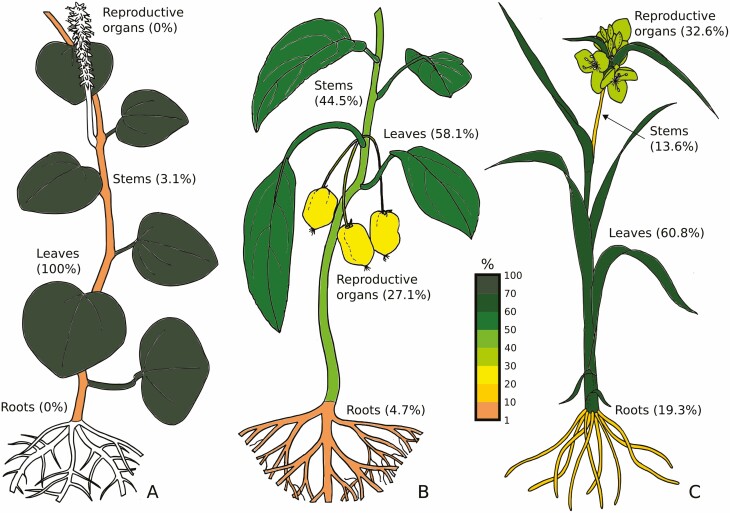
Diagram comparing the percentage presence of raphides found in each of the primary location categories, in Magnoliids (A), Eudicots (B) and Monocots (C). The colour scheme indicates the percentage of species that show evidence for raphide presence for each primary location; leaf, reproductive organ, stem and root. Based on the currently available data for each clade (32 Magnoliids, 226 Eudicots, 527 Monocots) summarized from **Supporting Information—Table S6**. Drawings by J. Nelson.

When summarizing the location information at the level of the major angiosperm clades (Magnoliids, Eudicots and Monocots), leaves are the most prominent raphide-containing primary location in all three clades (Fig. 10). However, Magnoliids show raphide presence in leaves in all studied species, with only one report of leaf and stem presence (*Piper caldense*). Conversely, Monocots show raphides in more than 50 % of the species analysed, and Eudicots in less than 50 % of species analysed. Moreover, reproductive organs show virtually the same prevalence between the two clades, but the percentage occurrence in Monocots is less than a third than the one in Eudicots.

## Discussion

Raphides and other CaOx crystals have been known for many years and have been isolated for a wide variety of reasons, such as to determine the acridity of the crystals (i.e. how they affect mucosa when ingested (Perera *et al.* 1990), to assess the defensive activity against herbivores (Konno *et al.* 2014), and to characterize them crystallographically (Jauregui-Zuniga *et al*. 2003). This review brings together all literature reporting CaOx raphides in plants and discusses the phylogenetic distribution of raphides across vascular plants and across plant organs. As the last raphide-focused review dates back to Franceschi and Horner (1980), this work offers a significant update of 317 additional research articles on the topic, broadening the view and settling the state-of-the-art on the subject.

### Phylogenetic distribution of raphides

CaOx raphides seem to be widely distributed across flowering plants (Fig. 8). Outside of flowering plants, we only found one fern (*Asplenium cardiophyllum*, Polypodiales), which contained raphides in the leaf epidermis. Ferns are a relatively minor food source for animals and therefore might not need raphides as a defence. They have been shown to be carcinogenic to humans (Alonso-Amelot and Avendaño 2002). Similarly, Balick *et al.* (1978) reported that ferns have toxic effects on both invertebrates and vertebrates due to the presence of compounds such as sesquiterpene lactones and ­cyanogenic glycosides, and that only arthropods have managed to overcome these defences. Furthermore, humid non-cultivated soils normally have low amounts of heavy metals (Franceschi and Nakata 2005), so sequestration of heavy metals via crystals is not necessary.

For the gymnosperms and early branching angiosperm lineages (Amborellales, Nymphaeales and Austrobaileyales) we did not recover any raphide-forming taxa in this review, although other crystals have been reported in these groups (Franceschi and Nakata 2005). The early branching angiosperm lineage magnoliids do contain raphide-forming taxa **[**see **Supporting Information—Table S3****]**. The order Piperales makes up for 97 % of the taxa reported for magnoliids. All 32 sampled species of Piperales were said to form raphides in the leaves (Horner *et al.* 2015). Members of this order are predominantly herbaceous, with few woody species, which explain the great abundance of raphides present in terms of herbivory defence (Thurston 1976; Korth *et al.* 2006). There was only a report on a single species in Magnoliales, *Magnolia sieboldii*, which was reported to contain no raphides (Zhang *et al.* 2014).

The remaining two major angiosperm clades are the monocots and eudicots. Monocotyledons, with 76.1 % of studied species showing raphides **[**see **Supporting Information—Table S3****]**, are the clade with the most extensive raphide presence (Fig. 8). Monocots include plants from all biogeographical and climatic regions of the world. As predominantly herbaceous, flowering plants, monocots’ first means of protection and defence against predators might be strongly linked to their ability to produce and distribute CaOx crystals within all anatomical locations, particularly leaves. For example, in *A. tequilana*, the defensive function of raphides is supported by reports of dermal irritation among workers in tequila distilleries and plantations upon contact with the stems and the extracts from the leaves (Salinas *et al.* 2001). Conversely, Arecales, alongside some Pandanaceae genera, are the main monocot taxa exhibiting anomalous secondary growth in the form of arboraceous or ‘woody’ stems, and are the order with the lowest percentage of raphide-containing monocot species. In Arecales, only 34.2 % of studied species showed raphides, with the majority in the reproductive organs (specifically embryos), as opposed to 70–100 % of studied species of the other non-woody monocot orders, such as Commelinales or Asparagales.

The early branching Eudicotyledon lineages (Ranunculales, Trichodendrales, Proteales and Buxales) seem to not have been purposefully examined for raphides. Only one report mentioned the Ranunculaceae species *Ranunculus apiifolius,* stating that it did not contain any crystals (Borrelli *et al.* 2011).

Within the core eudicots, Rosids constitute a large clade with very diverse features and distributions and are divided into three main groups: ‘core’ Rosids, Rosids I (Fabidae) and Rosids II (Malvidae). Rosids as a whole are only represented by 23 species with raphides **[**see **Supporting Information—Table S3****]**. Rosids tend to be non-woody and display a wide variety of morphologies. Species of the family Vitaceae, of the core Rosid group, are characterized by woody lianas that climb using leaf-opposed tendrils (Keller 2020), whereas Rosid I includes species such as willows, flax and violets (Malpighiales) and represents the smaller trees of the lowland tropical rainforest. Additionally, the genus *Euphorbia*, within the Malpighiales, includes succulents similar to cacti (Stevens 2016). This great diversity of life forms might explain the distribution of raphides over the phylogenetic tree and the various locations in the plant (see below).

The Superasterids include 16 orders, with Caryophyllales sister to the rest. Caryophyllales is represented in literature by seven families and a total of 264 species (high majority of Cactaceae) of which 38 species (14.4 %) have been reported to form CaOx raphides **[**see **Supporting Information—Table S3****]**. Caryophyllales are a varied order including trees, lianas, mangroves, herbaceous plants, succulents, and even insectivores. Their preferred environments are alkaline, arid and/or desert regions around the globe (Berry *et al.* 2018). The general lack of raphides could be compensated for by the presence of other defence mechanisms, such as spines, but also related to the abundance of calcium in the arid soils, which might remove the selective pressure to retain raphide-forming biochemical pathways (Frank 1972). The remaining Asterids include 273 studied species of which almost 70 % contain raphides **[**see **Supporting Information—Table S3**].

Overall, raphides appear to be more prevalent in some clades, particularly Monocots, Commelinids and Asterids I, while also sporadically found in other clades but with lesser occurrence in Rosids, core Asterids and Asterids II (Fig. 8). This might indicate that the capacity to form CaOx raphides may have evolved early in the history of vascular plants. The hypothesis that raphides as a morphotype may be specific to certain orders can be supported in light of the available data. Such specificity may be seen in the orders of Alismatales, Dioscoreales, Liliales and Asparagales within the Monocots, as well as Gentianales and Lamiales within the Asterids. Preference to raphide production might be related to function or location.

### Raphide distribution in plants organs

Next to the phylogenetic overview presented above, this study also inventoried the location of the plant organ where the raphides were found (i.e. leaf, reproductive organ, root and stem; Fig. 9). Within the leaves, the prominent tissues for raphides are the lamina parenchyma (mesophyll) and epidermis (Fig. 9A; **Supporting Information—Table S5**). Since leaves are a primary attraction for herbivores, these tissues represent ideal locations for the development of raphides, which would strengthen them and aid the protection of the plant through their needle shape (Thurston 1976; Korth *et al.* 2006).

Within the reproductive organs (Fig. 9A; **Supporting Information—Table S5**), the most common locations are the anthers and pollen grains in the androecium, and the ovaries and style in the gynoecium. Raphides in the androecium were reported more times than in the gynoecium. Protection of ovules, and consequently embryos, against attack or breakage is essential for the development of plants, thus the ability to produce defensive elements in female reproductive organs is of great importance. Nonetheless, dehiscence of pollen must occur first for fertilization to be successful, and the presence of CaOx crystals in the pollen-producing anthers and in pollen could be a defensive mechanism against consumption by pollinators (Barabe *et al.* 2004). However, the study responsible for finding raphides and other CaOx morphologies on anthers and pollen in members of Araceae noted that raphides are released onto the surface of the anther epidermis during the early stages of development, before dehiscence takes place (Barabe *et al.* 2004). It is unclear whether this is a necessary step or a consequence of pollen release. Moreover, since these organs are typically small and their structure is essential for successful pollination and fruit development, CaOx crystals here could simply be offering an additional structural support to delicate tissues.

Within stems, the most common raphide-containing secondary locations are the ground tissue, followed by cortex, parenchyma and tubers (Fig. 9A; **Supporting Information—Table S5**). Raphides in the ground tissue would provide advantageous additional protection (Ward *et al.* 1997; Ruiz *et al.* 2002), particularly against bigger herbivore; other research (Konni *et al.* 2014) suggests a link between raphide density in tubers with levels of acridity, and the possibility of the ‘needle effect’ facilitating carcinogenic substances to pass through cell barriers. More research regarding the size of the raphides in stems and leaves could provide a systematic approach to this information as a function of the plant environment, and support or reject these hypotheses.

Lastly, root tubers were the most prominent raphide-containing secondary location within the roots. Root tubers are swollen roots that accumulate and store water and food material for the plant. Raphide presence is suggested to protect the tubers in the same way raphides may deter predators in leaves (Sawidis *et al.* 2005). Raphides found in the roots might also be involved in the regulation of calcium levels by acting as calcium sinks, as reported in *Vitis berlandieri* (Storey *et al.* 2003), though the advantage of the raphide morphotype against other crystal morphologies for fast formation and dissolution has not been systematically addressed. It is also possible that the provided structural support could be an asset to the survival of the plant that produces them.

When comparing raphide prevalence across the three clades, Magnoliids, Eudicots and Monocots, it is clear to see that the leaves consistently show the highest percentage of raphide presence (Fig. 10). This is calculated by the number of unique species per clade showing a presence in leaves divided by the total unique species per clade. For example, all 32 Magnoliid species have raphides in their leaves, whilst only one species has raphides in the stem as well (Horner *et al.* 2015). Members of this clade are predominantly herbaceous, with few woody species, which positively correlates with the great abundance of raphide presence in the leaves. Whereas the total unique species is only 32 for Magnoliids, there were 236 Eudicots and 528 Monocots; a greater species representation. In Eudicots and Monocots, more than 50 % of species presented raphides in their leaves, 58.1 and 60.8 % respectively. Contrastingly, where Eudicots showed the second highest raphide prevalence in their stems (44.5 %), Monocots showed the lowest prevalence of raphides (13.6 %). The emerging trends from the ensemble of the results strongly suggests that the presence of raphides is a feature that is informative at the clade level and for taxonomic identification.

Overall, it was found that leaves are the predominant location for raphides, followed by stems, a result that generally supports the previous theories about raphide-mediated protection against herbivory. The fact that monocots, being largely herbaceous and prone to herbivory, are the clade with the highest number of sampled species and raphide-forming species, also supports this idea. This does not negate the possibility of raphides being involved in structural support in different locations and through different tissues of the plant or that the defensive effects are indirect and only serve mostly as herbivory deterrent (Paiva 2021). Therefore, the herbivory theory requires more experimental evidence (Paiva 2021) but this would require a more attentive investigation of the raphide occurrence, their size and their morphologies.

### Future outlook

It is clear that some clades have been more rigorously studied for raphide presence than others (Fig. 8) and that there is an under-representation of certain taxa. Only 9 out of the 69 listed orders have been considerably studied. Even then, only Acorales have been significantly represented, which is due to the order having only three members. Reports on Asparagales, Arecales and Caryophyllales are more common. Meanwhile, most of the remaining orders have less than 35 reported species and/or 1 % representation. Next to this, the absence of raphides is often not reported. Increased reporting of raphide presence and absence would greatly add to the knowledge of raphide phylogenetic distribution and occurrence in the plant. Details on morphologies and sizes of raphides would allow sorting into subtypes (following Raman *et al.* 2014), and would ascertain whether these variations are caused by genetic or environmental influences (Crowther 2009; Chairiyah *et al.* 2013). This additional level of detail could reveal new patterns that would provide taxonomically informative characters. Investigating raphide distribution in systematically sampled clades could help to answer questions on the evolutionary pressures acting on raphide formation and their morphological variants. This kind of phylogenetic overview would allow for genomic studies, which could pinpoint the set of genes that a plant must have to be able to biomineralize raphides. It should also reveal the mechanism of raphide formation and whether there are multiple ways that plants synthesize raphides or a single ancestral genetic toolkit that has been lost many times.

Being able to confirm evolutionary patterns would not only be a matter of fundamental interest. As it has been shown by Nakata (2012) that crystal formation can be engineered in a plant, and thus also potentially suppressed. Nakata (2012) found that in *Arabidopsis* sp. (Brassicaceae, Brassicales), which does not naturally produce CaOx crystals, the plant could be engineered to form druses and random CaOx crystal deposits by the introduction of genes encoding for oxalate production. Given the rising necessity for plants to be more resistant to a rapidly changing environment while reducing treatments that can disperse chemicals in the environment, raphide engineering could be an all-new approach to plants that are more structurally resistant and inherently protected from predators, as well as having improved photosynthetic adaptation and heavy metal tolerance.

## Conclusion

Our review reveals raphide occurrence has been studied in 33 orders, 76 families and 1305 species, with raphides presence confirmed in 24 orders, 46 families and 797 species. These taxa represented less than 1 % of known species per family, meaning that for many families, the frequency of raphide occurrence remains unknown. More reports of raphide occurrences are strongly encouraged by the authors, as several orders are currently too unrepresented to allow for grounded predictions or comparisons. It is encouraged to view those taxa without raphides reported as species that require further research rather than as conclusive non crystal-forming species.

Raphides occur most prominently in leaves but there are only few reports on raphides in roots. This fits with the idea that their primary function is for herbivory defence. However, not enough data are present to test this hypothesis and investigate the evolutionary pressures acting on raphide formation.

The overview provided encourages researchers to see raphides in plants not as a mere curiosity, but as a trait ripe with potential for advancement in taxonomy and phylogenetics, and for critically investigating adaptive evolution and biochemical pathways. Research aimed at characterizing new taxa and biochemical pathways for biomineralization are both encouraged and recommended. The insolubility of CaOx will lead inevitably (i.e. thermodynamically) to precipitation and crystal formation. The key botanical question is how the plant can direct the cellular factors that determine the shape in which this crystallization takes place. The collation of previous studies here lays the groundwork to unveil the genetic origin and evolution of raphides in plants, providing an approach to predict crystal presence in unreported taxa, while also highlighting knowledge gaps in understanding the presence and role of raphides in structures of plants.

This review, therefore, provides a guide for botanists who had previously not considered looking at raphides, allowing for planning and designing new experiments, and particularly for choosing appropriate species to sample. Even in the context of botanical studies with different targets, the unmistakable and peculiar morphology of raphides allows them to be easily recorded and reported with minimal additional effort to add a significant value to the field of biomineral research.

## Supporting Information

The following additional information is available in the online version of this article –

**Link S1. Reference list for Raphide presence**. Contains a link to the Dutch data-sharing academic platform (surfdrive), for a publicly accessible folder with Endnote and Mendley format of the reference library.

**Table S1. Raphide Presence, Location per species**. The excel spreadsheet contains a list of all species where raphides have been reported, with specifics on the location and full taxonomy. A separate sheet in the file contains the references.

**Table S2. Location per clade table**. The excel spreadsheet contains a table ordered by clade, order, family, and reports the raphide presence in primary and secondary locations, with numbered counts.

**Table S3. Raphide Presence Computing table**. The excel spreadsheet contains a table ordered by clade, order, family, and any calculation associated to the raphide presence reports.

**Table S4. All species per Clade table**. The excel spreadsheet contains a table with the full taxonomical identification of any species for which raphides have been reported in the literature.

**Table S5. Primary and secondary location specifics table**. The excel spreadsheet contains a table ordered by clade, order, family, and reports the raphide presence with details about the primary locations.

**Table S6. Primary location computing by clade table**. The excel spreadsheet contains a table ordered by clade, order, family, and all the calculation regarding raphide presence in different primary locations.

plad031_suppl_Supplementary_Material

## Data Availability

The data underlying this article are available in the article and in its online Supporting material. The bulk of the data is available in Excel format, which includes associated computations. Any request for different formats can be addressed to the corresponding author and will be readily acknowledged.

## Notes

**Form and Function. Chief Editor**: Kate McCulloh
